# Ginsenoside Re Protects against Serotonergic Behaviors Evoked by 2,5-Dimethoxy-4-iodo-amphetamine in Mice via Inhibition of PKCδ-Mediated Mitochondrial Dysfunction

**DOI:** 10.3390/ijms22137219

**Published:** 2021-07-05

**Authors:** Eun-Joo Shin, Ji Hoon Jeong, Bao-Trong Nguyen, Naveen Sharma, Seung-Yeol Nah, Yoon Hee Chung, Yi Lee, Jae Kyung Byun, Toshitaka Nabeshima, Sung Kwon Ko, Hyoung-Chun Kim

**Affiliations:** 1 Neuropsychopharmacology and Toxicology Program, College of Pharmacy, Kangwon National University, Chunchon 24341, Korea; shinej@kangwon.ac.kr (E.-J.S.); nguyenbaotrong.ydct@gmail.com (B.-T.N.); naveenbiochem07@gmail.com (N.S.); 2 Department of Global Innovative Drugs, Graduate School of Chung-Ang University, College of Medicine, Chung-Ang University, Seoul 06974, Korea; jhjeong3@cau.ac.kr; 3 Ginsentology Research Laboratory, Department of Physiology, College of Veterinary Medicine and Bio/Molecular Informatics Center, Konkuk University, Seoul 05029, Korea; synah@konkuk.ac.kr; 4 Department of Anatomy, College of Medicine, Chung-Ang University, Seoul 06974, Korea; yoonhee@cau.ac.kr; 5 Department of Industrial Plant Science & Technology, Chungbuk National University, Chungju 28644, Korea; leeyi22@hanmail.net; 6 Korea Society of Forest Environmental Research, Namyanju 12106, Korea; forbjk@hanmail.net; 7 Advanced Diagnostic System Research Laboratory, Fujita Health University Graduate School of Health Science, Toyoake 470-1192, Japan; tnabeshi@ccmfs.meijo-u.ac.jp; 8 Department of Oriental Medical Food and Nutrition, Semyung University, Jecheon 27136, Korea

**Keywords:** ginsenoside Re, 5-HT_2A_ receptor agonist DOI, serotonergic behaviors, head twitch response, hyperthermia, mitochondrial burden, hypothalamus, PKCδ knockout mice

## Abstract

It has been recognized that serotonin 2A receptor (5-HT_2A_) agonist 2,5-dimethoxy-4-iodo-amphetamine (DOI) impairs serotonergic homeostasis. However, the mechanism of DOI-induced serotonergic behaviors remains to be explored. Moreover, little is known about therapeutic interventions against serotonin syndrome, although evidence suggests that ginseng might possess modulating effects on the serotonin system. As ginsenoside Re (GRe) is well-known as a novel antioxidant in the nervous system, we investigated whether GRe modulates 5-HT_2A_ receptor agonist DOI-induced serotonin impairments. We proposed that protein kinase Cδ (PKCδ) mediates serotonergic impairments. Treatment with GRe or 5-HT_2A_ receptor antagonist MDL11939 significantly attenuated DOI-induced serotonergic behaviors (i.e., overall serotonergic syndrome behaviors, head twitch response, hyperthermia) by inhibiting mitochondrial translocation of PKCδ, reducing mitochondrial glutathione peroxidase activity, mitochondrial dysfunction, and mitochondrial oxidative stress in wild-type mice. These attenuations were in line with those observed upon PKCδ inhibition (i.e., pharmacologic inhibitor rottlerin or PKCδ knockout mice). Furthermore, GRe was not further implicated in attenuation mediated by PKCδ knockout in mice. Our results suggest that PKCδ is a therapeutic target for GRe against serotonergic behaviors induced by DOI.

## 1. Introduction

Ginseng (*Panax ginseng*) is a naturally occurring herb. Commercially available ginseng formulations are mainly extracted from the roots of ginseng plants [1]. Ginsenosides, a form of triterpene glycosides (saponins), are the major bioactive ingredients in ginseng. Importantly, it has been demonstrated that ginsenosides enhance brain function via antioxidative and antineuroinflammatory activities. In addition, they slow down or attenuate numerous neurodegenerative and psychiatric disorders. There are approximately 150 ginsenosides, and among them, 40 have been found in *Panax ginseng* per se [2,3]. In particular, ginsenoside Re (GRe) is the main ginsenoside that has shown potential neuroprotective activities [4]. Although mostly the root of the ginseng plant has been used in herbal medicine, earlier studies have reported that the main bioactive component of ginseng (i.e., GRe) is more abundantly present in berries, flower buds, and leaves than in the roots [4,5,6,7,8], indicating the important pharmacoeconomical intervention of GRe for developing naturally occurring drug resources. Therefore, in our previous studies, we have investigated the neuropsychoprotective activities of GRe [9,10,11,12,13].

Serotonin syndrome is a severe hazardous condition that can be life-threatening [14], and the syndrome includes altered mental status, autonomic responses, and the triad of motor symptoms [15,16]. Excessive serotonin release and consequent overactivation of central and peripheral serotonin receptors are known to cause serotonin syndrome [14]. For instance, abuse of serotonergic compounds, such as methylenedioxymethamphetamine (MDMA) [17,18,19,20,21,22], 5-methoxy-N,N-dimethyltryptamine (5-MeO-DMT) [23], and psilocybin [24], or dissociative drugs, such as dextromethorphan [25,26,27,28], has been reported to provoke serotonin syndrome in humans. Indeed, it has been reported that frequent usage of antidepressants, such as monoamine oxidase (MAO) inhibitors and selective serotonin reuptake inhibitors (SSRIs), enhances the risk of serotonin syndrome [14,29]. Therefore, the fact that sustained exposure to antidepressant drugs presents a potential for inducing serotonin syndrome is of concern. 

For example, fluoxetine, an SSRI, has various effects on energy metabolism in the hepatocellular mitochondria of rats, and it is potentially toxic at high doses [30]. It affects apoptosis by increasing the voltage sensitivity of the mitochondrial voltage-dependent anion channel [31]. Fluoxetine induces the inhibition of oxidative phosphorylation and decreases mitochondrial ATP synthase activity in the rat brain [32]. Exposure to norfluoxetine, an active metabolite of fluoxetine, reduces the membrane potential and activity of mitochondrial complexes and leads to apoptotic changes [33]. However, it is unclear whether mitochondrial dysfunction is involved in serotonin syndrome behaviors.

Among serotonin receptors, post-synaptic 5-HT_1A_ and 5-HT_2A_ receptors have been suggested to be importantly involved in serotonin syndrome [14,34]. Consistently, it has been reported that the specific 5-HT_1A_ receptor agonist 8-OH-DPAT or the specific 5-HT_2A_ receptor agonist (4-Bromo-3,6-dimethoxybenzocyclobuten-1-yl)methylamine hydrobromide TCB-2 induces serotonin syndrome behavior in mice [35,36,37]. In most cells and tissues, 5-HT receptors activate phospholipase C, which causes stimulation of protein kinase C (PKC) [38]. Among PKC isozymes, PKCδ is mainly involved in several cellular transduction pathways coupled with oxidative stress, inflammation, and cell death [39,40,41,42,43,44,45]. We [13,41,42,43,44,45,46,47,48,49] and others [50,51,52,53] have suggested that PKC is important for neuropsychotoxicity induced by amphetamine and its analogues. In particular, the PKCδ gene is a crucial target for generating serotonergic behaviors [35,54].

We have recently filed a patent (patent number KR 1-1-2020-1158807-40: The pharmaceutical composition for the prevention and treatment of serotonin syndrome behaviors). In this study, we observed that protopananxatriol saponins (PPT) blocked serotonergic behaviors in mice and that the GRe in PPT played a major role in attenuating against serotonin syndrome behaviors. Thus, in the present study, we attempted to extend our knowledge of the GRe-mediated pharmacological mechanism against the serotonergic behaviors induced by 2,5-dimethoxy-4-iodo-amphetamine (DOI), an amphetamine analog and a 5-HT_2A_ receptor agonist. We investigated the following: (1) whether DOI-mediated 5-HT_2A_ receptor stimulation induces mitochondrial dysfunction and the consequent oxidative burden; (2) whether PKCδ activation is involved in DOI-induced serotonergic changes, mitochondrial burden, and serotonergic behaviors using pharmacological and genetic inhibition of PKCδ; (3) whether GRe affects DOI-induced neuronal and behavioral changes; and (4) whether GRe modulates its effects by affecting PKCδ activation and consequent changes after DOI treatment. Since our recent studies have showed that the serotonergic impairment in the hypothalamus is much more pronounced than in the hippocampus or in the prefrontal cortex in several serotonin syndrome models [38,39,40], we have focused on the hypothalamic changes in the present study. We observed that GRe attenuated DOI-induced serotonin syndrome behaviors by inhibiting mitochondrial translocation of PKCδ, mitochondrial dysfunction, and mitochondrial oxidative damage, and impaired enzymatic antioxidant systems in the hypothalami of mice. Consistently, PKCδ knockout (PKCδ KO) attenuated against these neurobehavioral impairments caused by DOI in mice. 

## 2. Results

### 2.1. Effect of GRe, Rottlerin, or MDL11939 (MDL) on the Mitochondrial Translocation of PKCδ Induced by DOI in the Wild-Type Mice

**Figure 1:**
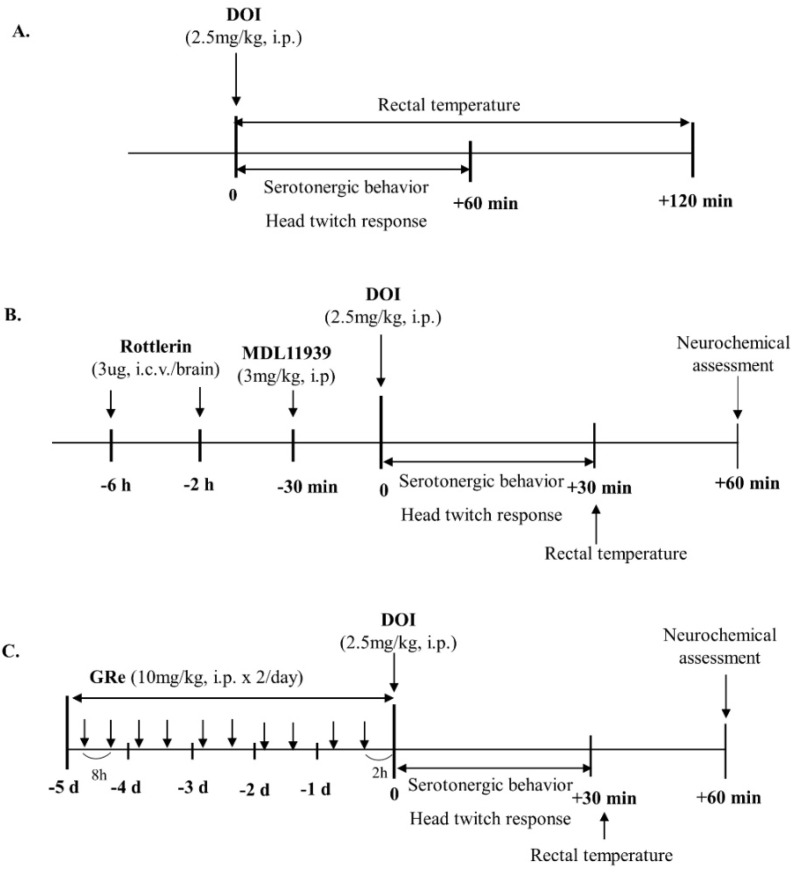
Schematic representation of experimental protocol for investigating the effect of GRe on the serotonergic behaviors induced by DOI in wild-type and PKCδ KO mice. DOI-induced serotonin syndrome behaviors and head twitch responses were analyzed every 6 min for the first 1 h, and rectal temperature was estimated every 15 min for 2 h (the first 1 h plus the second 1 h) (**A**). Rottlerin was given (3 µg, i.c.v./brain) 6 h and 2 h before the DOI administration. MDL11939 (3 mg/kg, i.p.) was given 30 min prior to DOI injection (**B**). GRe (10 mg/kg, i.p.) was injected twice a day at an 8 h interval for 5 consecutive days. DOI was injected 2 h after the final administration of GRe (**C**). Mice were sacrificed 1 h post-DOI-administration for assessing neurochemical changes (**B**,**C**). GRe, ginsenoside Re; DOI, 2,5-dimethoxy-4-iodo-amphetamine.

**Figure 2:**
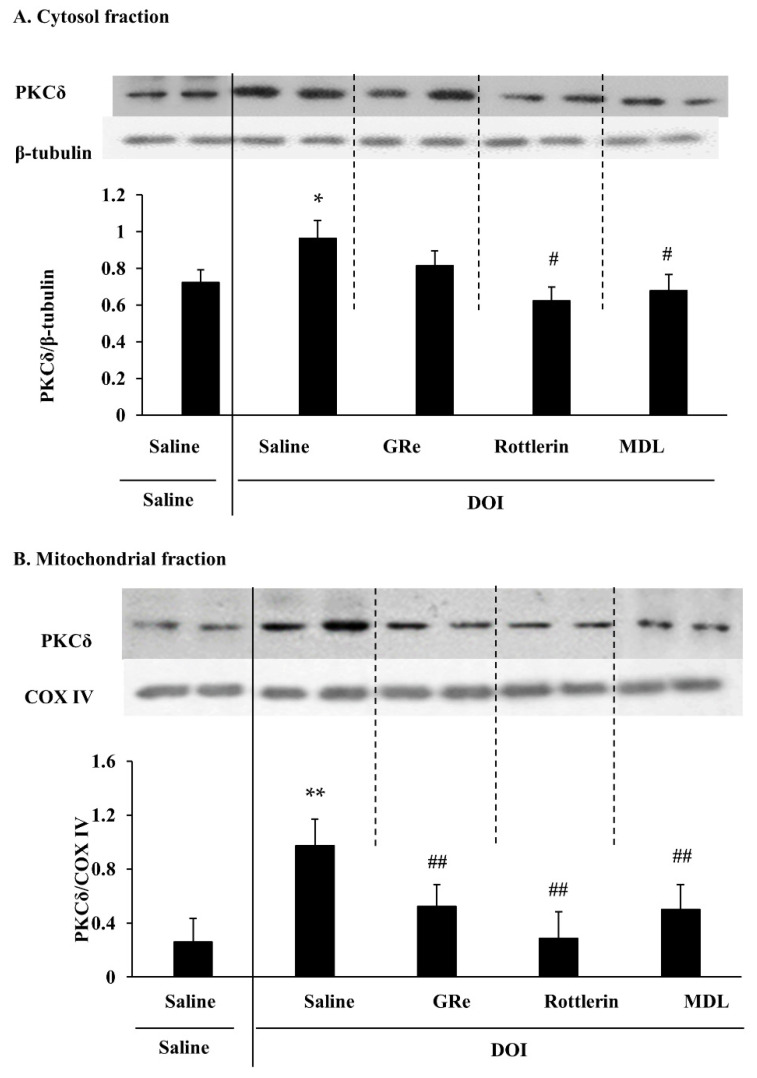
Effect of GRe, rottlerin, or MDL on the cytosolic (**A**) and mitochondrial (**B**) expression of PKCδ 60 min after the DOI administration in the hypothalamus of wild-type mice. Data are expressed as the mean ± SEM (4–6 animals). DOI, 2,5-dimethoxy-4-iodo-amphetamine; MDL, MDL11939; GRe, ginsenoside Re. * *p* < 0.05, ** *p* < 0.01 vs. saline/saline. ^#^ *p* < 0.05, ^##^ *p* < 0.01 vs. saline/DOI (one-way ANOVA was performed for statistical analysis followed by Fisher’s LSD pairwise comparisons). For more details refer to Supplementary Figure S1.

We recently demonstrated that hypothalamic PKCδ levels might play a critical role in inducing serotonin behaviors [35,54,55]. As shown in Figure 1 of the experimental design, we investigated whether GRe, the PKCδ inhibitor rottlerin, or the 5-HT_2A_ receptor antagonist MDL affects the mitochondrial translocation of PKCδ induced by the 5-HT_2A_ agonist DOI. As shown in Figure 2, DOI treatment significantly increased cytosolic PKCδ (*p* < 0.05 vs. saline/saline; Figure 2A) and mitochondrial PKCδ (*p* < 0.01 vs. saline/saline; Figure 2B) in wild-type mice. The effect of DOI seemed more evident in the mitochondrial fraction than the cytosolic fraction. This increase was significantly inhibited (*p* < 0.01 vs. saline/DOI) by GRe, rottlerin, or MDL, and the inhibition by GRe or MDL paralleled that by rottlerin (Figure 2B).

### 2.2. Effect of GRe or MDL11939 (MDL) on the Alterations in Mitochondrial Membrane Potential and Intra-Mitochondrial Ca^2+^ Level Elicited by DOI in the Wild-Type and PKCδ KO Mice 

Earlier reports have indicated that DOI facilitated mitochondrial dysfunction [56]. We [10,12,13,57] and other researchers [58,59,60] have demonstrated that GRe attenuates mitochondrial dysfunction induced by neurotoxic insults. In addition, we have also reported that GRe attenuates mitochondrial dysfunction induced by dopaminergic insult via genetic and pharmacological inhibition of PKCδ [12,13]. Consistently, we observed here that DOI remarkably augmented the mitochondrial translocation of PKCδ (Figure 2B). Thus, we investigated whether GRe or PKCδ inhibition modulates the mitochondrial membrane potential and intramitochondrial Ca^2+^ levels induced by DOI. In addition, we examined the effect of 5-HT_2A_ antagonism in our experimental conditions. 

**Figure 3:**
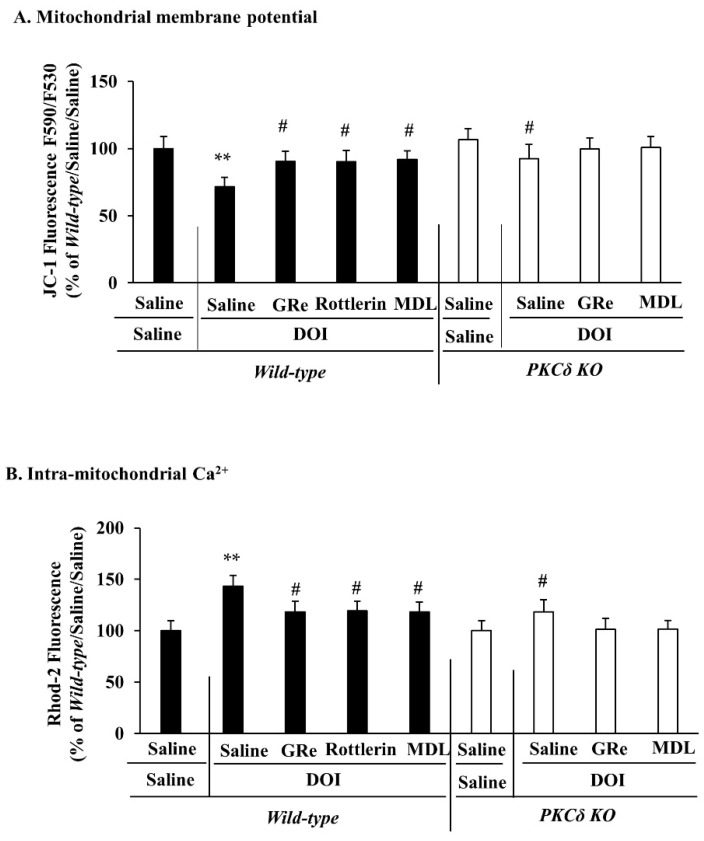
Effect of GRe or MDL on the alterations in mitochondrial membrane potential (**A**) and intra-mitochondrial Ca^2+^ level (**B**) induced by DOI in the hypothalamus of wild-type and PKCδ KO mice. Data are expressed as the mean ± SEM (6 animals/group). DOI, 2,5-dimethoxy-4-iodo-amphetamine; MDL, MDL11939; GRe, ginsenoside Re; PKCδ KO, PKCδ knockout mice. ** *p* < 0.01 vs. saline/saline/wild-type. ^#^ *p* < 0.05 vs. saline/DOI/wild-type (one-way ANOVA was performed for statistical analysis followed by Fisher’s LSD pairwise comparisons).

As shown in Figure 3A, DOI caused a significant decrease (*p* < 0.05 vs. saline/saline) in the mitochondrial membrane potential in the wild-type mice, whereas genetic depletion of PKCδ significantly attenuated (*p* < 0.05 vs. saline/DOI/wild-type) the decrease caused by DOI. Treatment with GRe, rottlerin, or MDL significantly inhibited (*p* < 0.05 vs. saline/DOI) DOI-induced decrease in the mitochondrial membrane potential in wild-type mice. However, neither GRe nor MDL affected PKCδ KO-mediated attenuation (Figure 3A). 

As shown in Figure 3B, DOI significantly increased (*p* < 0.05 vs. saline/saline) intra-mitochondrial Ca^2+^ accumulation, this increase was significantly alleviated (*p* < 0.05 vs. saline/DOI) by GRe, rottlerin, or MDL in the wild-type mice. PKCδ KO also significantly attenuated (*p* < 0.05 vs. saline/DOI/wild-type) the DOI-induced increase in intra-mitochondrial Ca^2+^ levels in mice. However, neither GRe nor MDL significantly altered the PKCδ KO-mediated attenuation (Figure 3B). 

### 2.3. Effect of GRe or MDL11939 (MDL) on the Alterations in the Mitochondrial Complex I and Complex II Activities Caused by DOI in Wild-Type and PKCδ KO Mice

**Figure 4:**
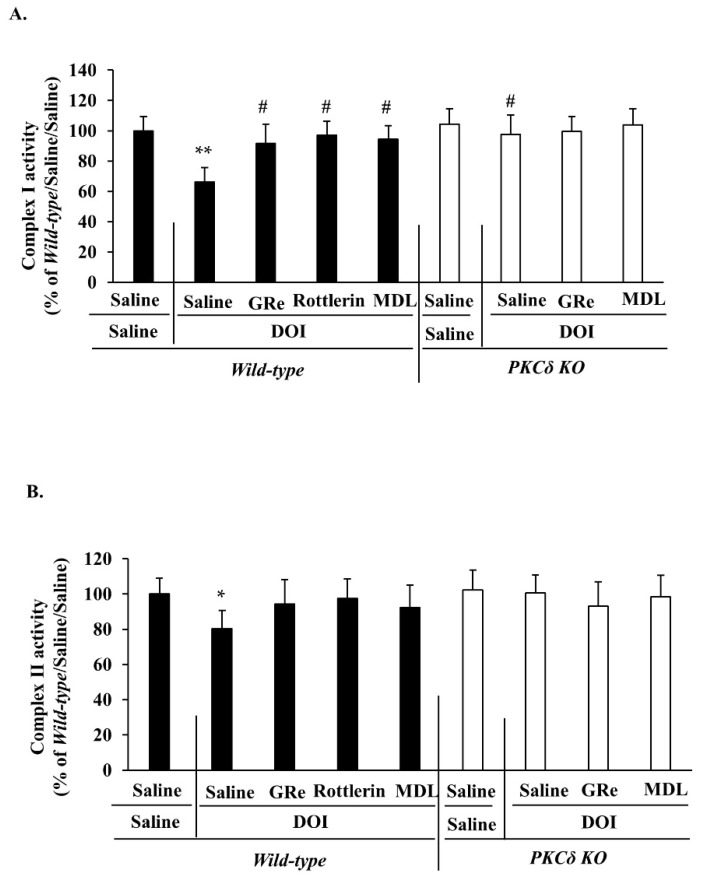
Effect of GRe or MDL on the changes in mitochondrial complex I (**A**) and complex II (**B**) activities caused by DOI in the hypothalamus of wild-type and PKCδ KO mice. Data are expressed as the mean ± SEM (6 animals/group). DOI, 2,5-dimethoxy-4-iodo-amphetamine; MDL, MDL11939; GRe, ginsenoside Re; PKCδ KO, PKCδ knockout mice. * *p* < 0.05, ** *p* < 0.01 vs. saline/saline/wild-type. ^#^ *p* < 0.05 vs. saline/DOI/wild-type (one-way ANOVA was performed for statistical analysis followed by Fisher’s LSD pairwise comparisons).

DOI treatment significantly decreased complex I (*p* < 0.01 vs. saline/saline; Figure 4A) and complex II (*p* < 0.05 vs. saline/saline; Figure 4B) activities in the wild-type mice. These effects were significantly attenuated (*p* < 0.05 vs. saline/DOI/wild-type) by PKCδ KO in mice. Treatment with GRe, PKCδ inhibitor rottlerin, or MDL resulted in a significant attenuation (*p* < 0.05 vs. saline/DOI) of the DOI-induced decrease in complex I activity in the wild-type mice. GRe, rottlerin, or MDL appeared to attenuate this effect without reaching statistical significance. In addition, GRe or MDL did not significantly alter PKCδ KO-mediated potentials against mitochondrial complex I and II activities caused by DOI (Figure 4).

### 2.4. Effect of GRe or MDL11939 (MDL) on DOI-Induced Oxidative Stress in the Mitochondrial and Cytosolic Fractions of the Hypothalamus of Wild-Type and PKCδ KO Mice 

**Figure 5:**
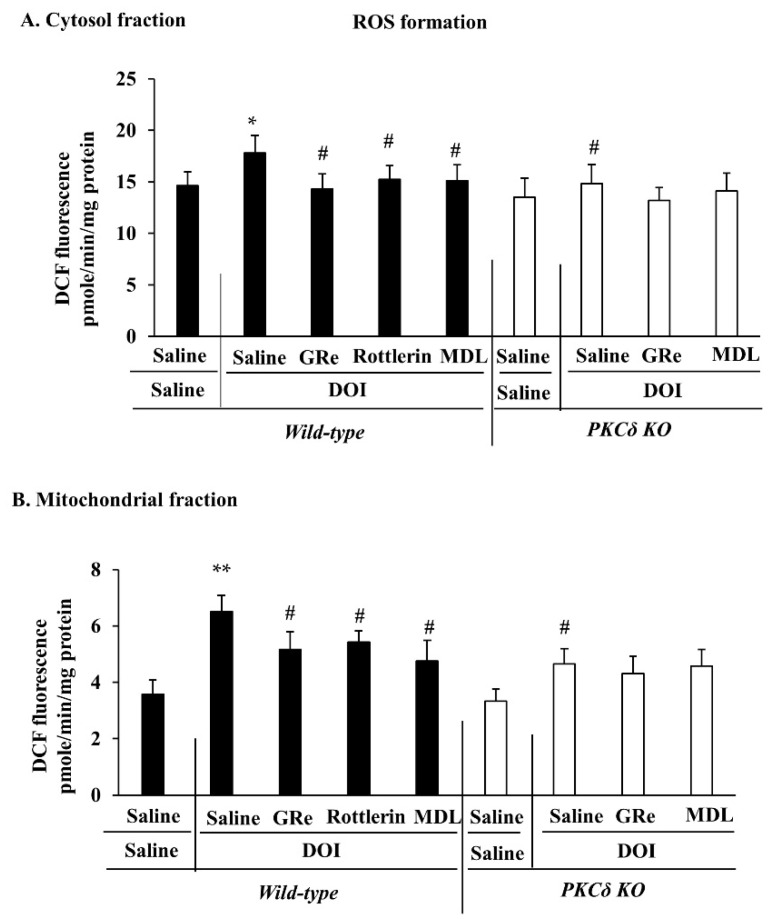

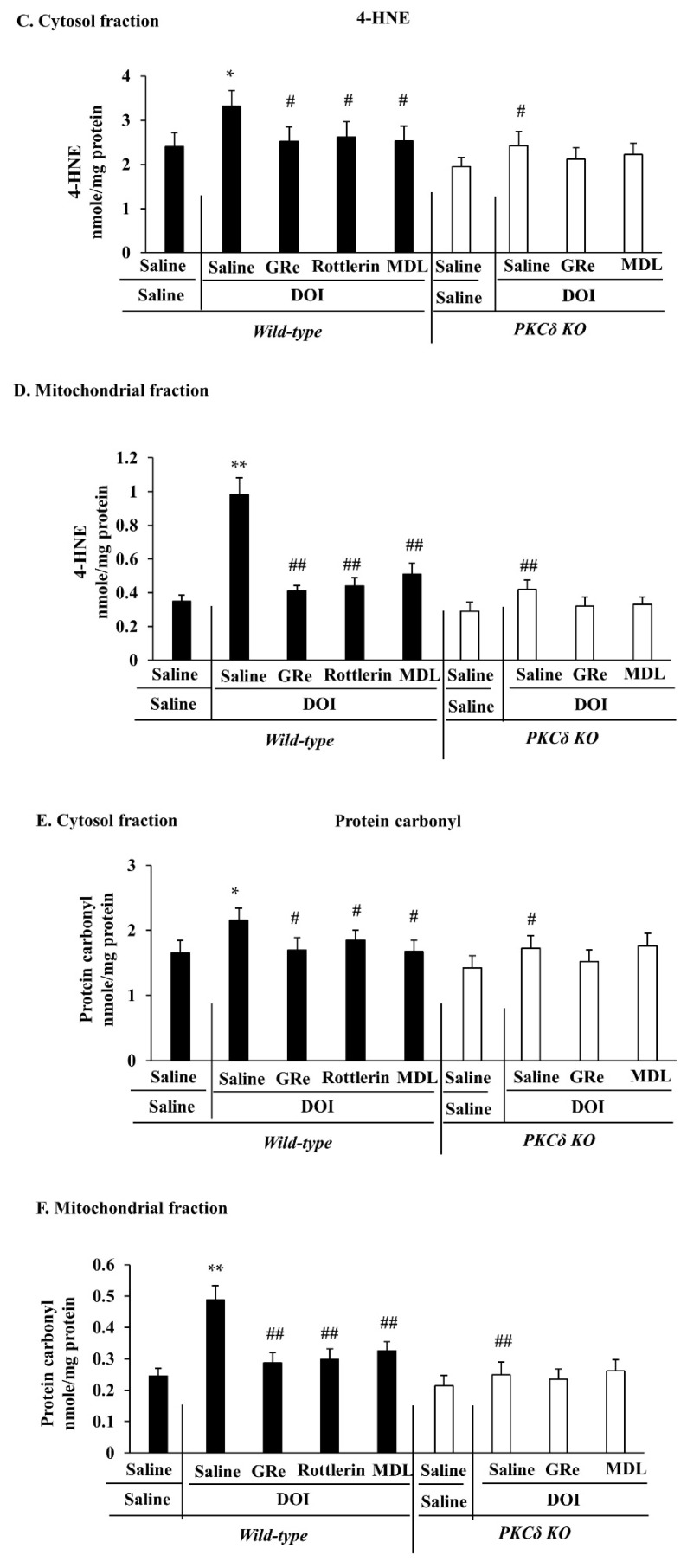
Effect of GRe or MDL on the DOI-induced oxidative stress in the cytosolic (**A**,**C**,**E**) and mitochondrial (**B**,**D**,**F**) fractions in the hypothalamus of wild-type and PKCδ KO mice. DOI-induced oxidative stress was assessed by ROS (**A**,**B**), 4-HNE (**C**,**D**), and protein carbonyl (**E**,**F**) levels. DOI, 2,5-dimethoxy-4-iodo-amphetamine; MDL, MDL11939; GRe, ginsenoside Re; PKCδ KO, PKCδ knockout mice. Data are expressed as the mean ± SEM (6 animals/group). * *p* < 0.05, ** *p* < 0.01 vs. saline/saline/wild-type. ^#^ *p* < 0.05, ^##^ *p* < 0.01 vs. saline/DOI/wild-type (one-way ANOVA was performed for statistical analysis followed by Fisher’s LSD pairwise comparisons).

We have recently demonstrated that 5-HT receptors require PKCδ to induce serotonergic behaviors and that PKCδ is an important factor for the harmful oxidant generation [35,54]. However, it remains to be elucidated whether the mitochondrial oxidative burden is involved in serotonergic behaviors. In this study, we found that DOI-induced oxidative stress appeared to be more evident in the mitochondrial fraction than in the cytosolic fraction, suggesting that mitochondria are more susceptible to DOI insult than cytosol. As shown in Figure 5, oxidative parameters (reactive oxygen species (ROS) formation, 4-hydroxynonenal (HNE), and protein carbonyl) in the mitochondrial and cytosolic fractions were significantly increased (cytosolic level of ROS, HNE, or protein carbonyl: *p* < 0.05 vs. saline/saline; mitochondrial level of ROS, HNE, or protein carbonyl: *p* < 0.01 vs. saline/saline) 1 h post-DOI treatment in wild-type mice. 

DOI-induced increases in oxidative parameters were significantly attenuated by PKCδ KO in mice (cytosolic ROS, HNE, or protein carbonyl: *p* < 0.05 vs. saline/DOI wild-type; mitochondrial ROS, HNE, or protein carbonyl: *p* < 0.01 vs. saline/DOI/wild-type). GRe, rottlerin, or MDL significantly inhibited DOI-induced oxidative damage (cytosolic ROS, HNE, or protein carbonyl: *p* < 0.05 vs. saline/DOI; mitochondrial ROS, HNE, or protein carbonyl: *p* < 0.01 vs. saline/DOI) in either fraction of wild-type mice. However, in the presence of DOI, GRe or MDL did not significantly affect antioxidant activity afforded by PKCδ KO in mice (Figure 5).

### 2.5. Effect of GRe or MDL11939 (MDL) on Changes in the Mitochondrial and Cytosolic Activities of Superoxide Dismutase (SOD) and Glutathione Peroxidase (GPx) Induced by DOI in Wild-Type and PKCδ KO Mice

It has been well-recognized that enzymatic antioxidants such as SODs, catalase, and peroxidases (i.e., GPx) are oxyradical scavengers; SOD catalyzes the dismutation of O_2_ (superoxide anion) to produce hydrogen peroxide (H_2_O_2_), and then catalase or GPx might catalyze the reduction of H_2_O_2_ to water. However, it is also apparent that in most eukaryotic cells, catalase is restricted to isolated compartments such as peroxisomes [61,62,63]; in particular, SOD and peroxidases (mainly GPx) are located in the cytoplasm and mitochondria of the cells [63,64,65,66]. Here, we asked whether GRe or genetic/pharmacological inhibition of PKCδ or MDL modulates SOD and GPx activities induced by DOI. 

**Figure 6:**
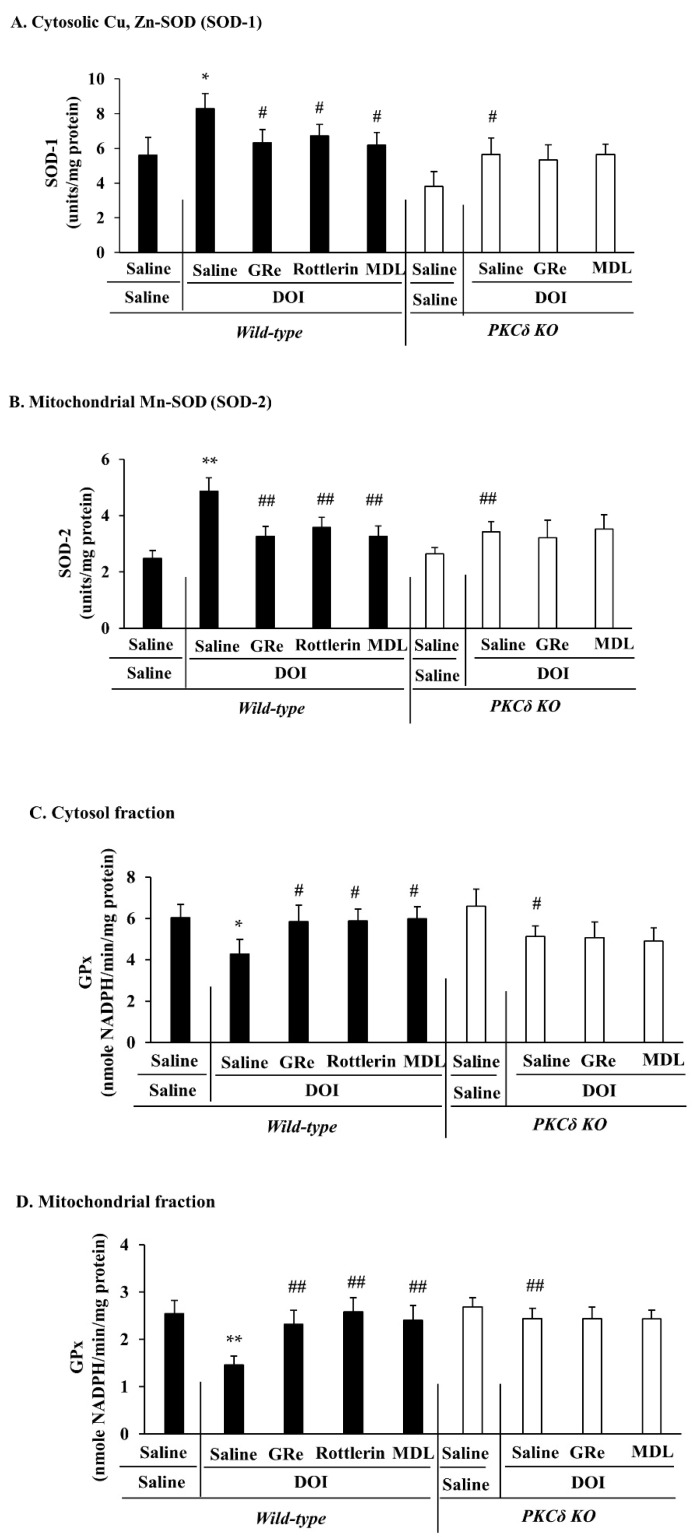
Effect of GRe or MDL on the changes in the activities of cytosolic Cu, Zn-SOD (SOD-1; (**A**)), mitochondrial Mn-SOD (SOD-2; (**B**)), cytosolic GPx (**C**), and mitochondrial GPx (**D**) induced by DOI in the hypothalamus of wild-type and PKCδ KO mice. DOI, 2,5-dimethoxy-4-iodo-amphetamine; MDL, MDL11939; GRe, ginsenoside Re; PKCδ KO, PKCδ knockout mice. Data are expressed as the mean ± SEM (6 animals/group). * *p* < 0.05, ** *p* < 0.01 vs. saline/saline/wild-type. ^#^ *p* < 0.05, ^##^ *p* < 0.01 vs. saline/DOI/wild-type (one-way ANOVA was performed for statistical analysis followed by Fisher’s LSD pairwise comparisons).

As shown in Figure 6, DOI significantly increased cytosolic SOD (SOD-1) (*p* < 0.05 vs. saline/saline) and mitochondrial SOD (SOD-2) activities (*p* < 0.01 vs. saline/saline) 60 min post-treatment in wild-type mice (Figure 6A,B). At that time, cytosolic and mitochondrial GPx activities were significantly decreased (cytosolic and mitochondrial fractions, *p* < 0.05 and *p* < 0.01 vs. saline/saline, respectively) in the presence of DOI in wild-type mice (Figure 6C,D). 

GRe, rottlerin, or MDL significantly inhibited DOI-induced increases in cytosolic SOD-1 (DOI plus GRe, rottlerin, or MDL: *p* < 0.05 vs. saline/DOI) and mitochondrial SOD-2 (DOI plus GRe, rottlerin, or MDL: *p* < 0.01 vs. saline/DOI) activities (Figure 6A,B). Consistently, either one significantly attenuated decreases in cytosolic (DOI plus GRe, rottlerin, or MDL: *p* < 0.05 vs. saline/DOI) and mitochondrial (DOI plus GRe, rottlerin, or MDL: *p* < 0.01 vs. saline/DOI) GPx activities from DOI insult in wild-type mice (Figure 6C,D). We observed that mitochondrial enzymatic antioxidants appeared to be more susceptible to DOI burden than cytosolic enzymatic antioxidants. Efficacy of GRe, rottlerin, or MDL appeared to be selective in the mitochondrial fraction (>cytosolic fraction). 

PKCδ KO also significantly attenuated DOI-induced changes in SOD-1 (*p* < 0.05 vs. saline/DOI/wild-type), SOD-2 (*p* < 0.01 vs. saline/DOI/wild-type), cytosolic GPx (*p* < 0.05 vs. saline/DOI/wild-type), and mitochondrial GPx (*p* < 0.01 vs. saline/DOI/wild-type) activities in mice. However, neither GRe nor MDL altered PKCδ KO-mediated attenuation of DOI-induced deregulation of cytosolic and mitochondrial SOD and GPx activities in mice (Figure 6A–D), suggesting that PKCδ is a critical target of antioxidant potential exhibited by GRe or MDL.

### 2.6. Effect of GRe or MDL11939 (MDL) against Serotonergic Behaaviors, Head Twitch Response, and Hyperthermia Caused by DOI in Wild-Type and PKCΔ KO Mice 

**Figure 7:**
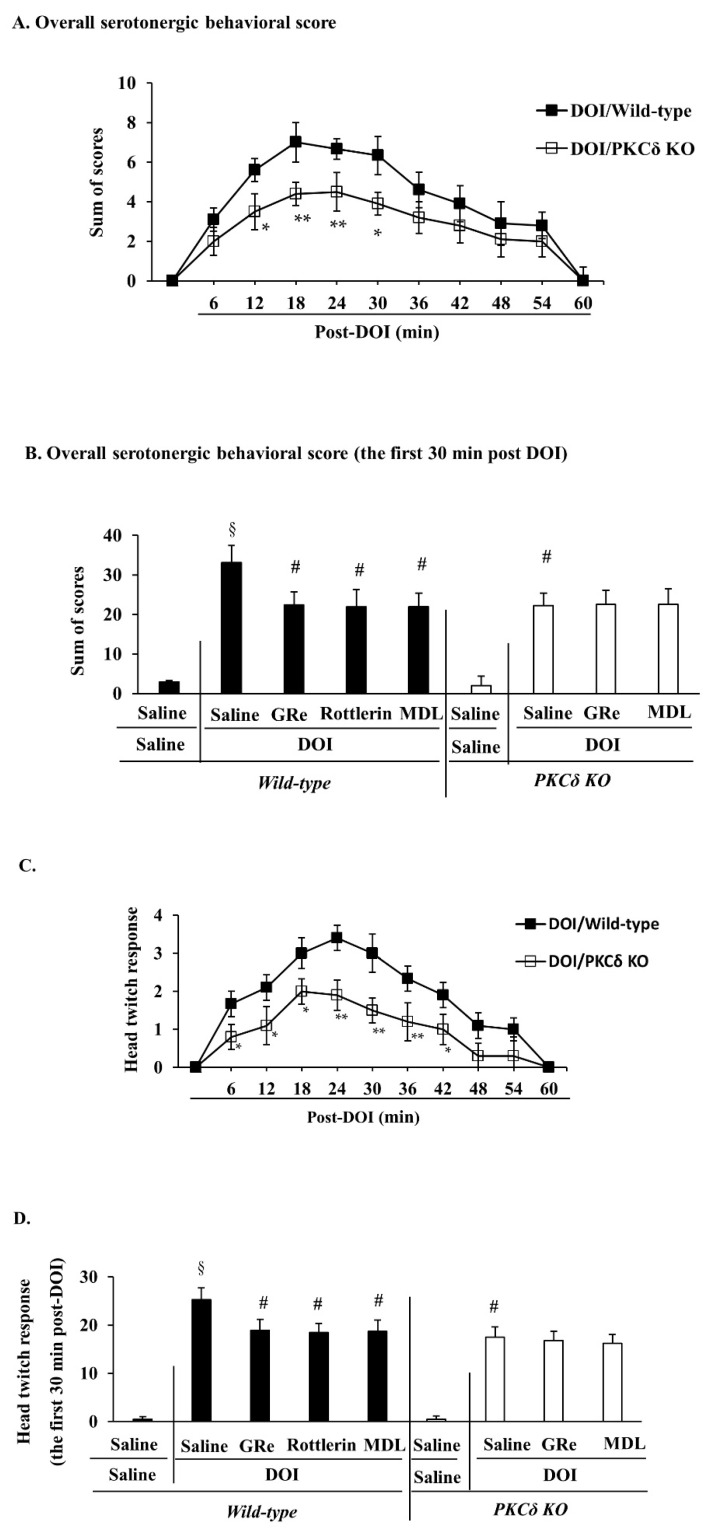

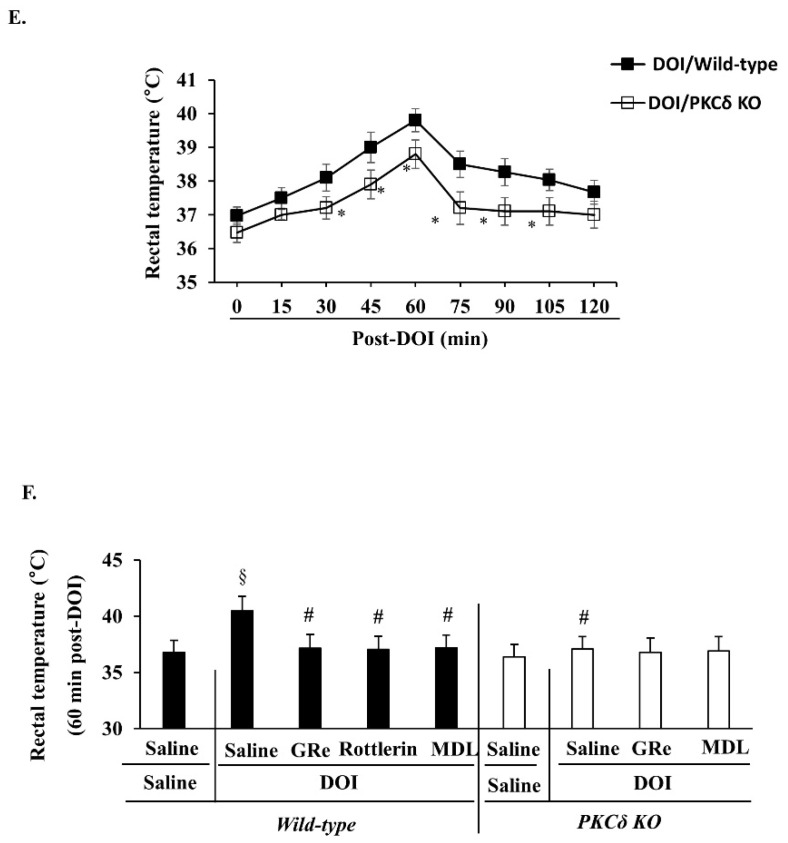
Effect of GRe or MDL on DOI-induced overall serotonergic behavioral score, head twitch response, and changes in rectal temperature in wild-type and PKCδ KO mice. Time course of changes in overall serotonergic behavioral score (**A**). Effect of GRe or MDL on DOI-induced serotonergic behavior within the first 30 min (**B**). Time course of changes in head twitch response (**C**). Effect of GRe or MDL on DOI-induced head twitch response within the first 30 min (**D**). Time course of changes in rectal temperature (**E**). Effect of GRe or MDL on hyperthermia 60 min post-DOI. Data are expressed as the mean ± SEM (6 animals/group). GRe, ginsenoside Re; MDL, MDL11939; DOI, 2,5-dimethoxy-4-iodo-amphetamine; PKCδ KO, PKCδ knockout mice. ** *p* < 0.01 vs. saline/saline/wild-type. ^§^ *p* < 0.01 vs. saline/saline/wild-type. ^#^ *p* < 0.05 vs. DOI/wild-type or saline/DOI/wild-type. Two-way ANOVA (**A**,**C**,**E**) or one-way ANOVA (**B**,**D**,**F**) was performed for statistical analysis followed by Fisher’s LSD pairwise comparisons. For more details refer to Supplementary Figure S2.

Treatment with DOI resulted in significant hyperthermia, complex serotonergic behaviors (i.e., hind limb abduction, forepaw treading, straub tail, low body posture, lateral head weaving, and tremor; Supplementary Figure S2), and head twitch response in wild-type mice. PKCδ KO significantly attenuated DOI-induced overall serotonergic behavioral scores (12 min and 30 min post-DOI administration: *p* < 0.05 vs. DOI/wild-type; 18 min and 24 min post-DOI administration: *p* < 0.01 vs. DOI/wild-type) and head twitch response number (6 min, 12 min, 18 min, and 42 min post-DOI administration: *p* < 0.05 vs. DOI/wild-type; 24 min, 30 min, and 36 min post-DOI administration: *p* < 0.01 vs. DOI/wild-type), and hyperthermia (30 min, 45 min, 60 min, 75 min, 90 min, and 105 min post-DOI administration: *p* < 0.05 vs. DOI/wild-type) (Figure 7A,C,E). 

Because DOI-induced serotonergic behavioral scores and head twitch response were more prominent in the first 30 min than in the second 30 min post-treatment, the effects of GRe, rottlerin, or MDL on the alterations in serotonergic behavioral score and head twitch response were evaluated during the first 30 min. In addition, the effect of GRe, rottlerin, or MDL on hyperthermia was examined 60 min post-DOI administration because hyperthermia was most evident at that time.

As shown in Figure 7B,D,F, GRe, rottlerin, or MDL significantly alleviated the overall serotoninergic behavioral score (*p* < 0.05 vs. saline/DOI), head twitch response (*p* < 0.05 vs. saline/DOI), and hyperthermia (*p* < 0.05 vs. saline/DOI) induced by DOI in wild-type mice. Consistently, PKCδ KO significantly mitigated the overall serotonergic behavioral score (*p* < 0.05 vs. saline/DOI/wild-type), head twitch response (*p* < 0.05 vs. saline/DOI/wild-type), and hyperthermia (*p* < 0.05 vs. saline/DOI/wild-type) in mice. The effects of PKCδ KO were comparable to those of rottlerin. In addition, neither GRe nor MDL had significant additional effects on PKCδ KO-mediated attenuation of the DOI-induced behavioral toxicity in mice. 

## 3. Discussion 

It is known that PKC is a critical molecular factor in the regulation of 5-HT receptors [67,68]. The 5-HT2 receptor family is related to the phosphorylation of PKC isozymes [69]. Moreover, PKC modulation is required for the internalization of 5-HT_2A_ receptor [70,71]. There is evidence that 5-HT_1A_ and 5-HT_2A_ receptors are mainly responsible for developing serotonin syndrome [35,54,55,72]. Other studies also have provided the insight that the 5-HT_2A_ receptor is closely associated with serotonergic behavioral responses [13,73]. 

We recently demonstrated that PKCδ might mediate serotonergic syndrome behaviors [35,54,55]. We also indicated that the neuroprotective effects mediated by ginsenoside are due to their antioxidant activity, mainly by alleviating synaptosomal/mitochondrial oxidative stress and mitochondrial dysfunction [10,12,13,42,74]. Similarly, the observations of the present study show that DOI-induced serotonergic behaviors are elicited mainly by mitochondrial oxidative stress, impaired enzymatic antioxidant system (i.e., reduced mitochondrial activity of GPx), mitochondrial translocation of PKCδ, decreases in mitochondrial transmembrane potential and mitochondrial complex (I > II) activity, and increased level of intra-mitochondrial Ca^2+^; GRe or 5-HT_2A_ receptor antagonist MDL inhibits these morbid scenarios by inhibiting PKCδ.

Mitochondria are susceptible to oxidant, pro-inflammatory, and pro-apoptotic effects [75,76]. It is well-known that mitochondrial disturbances, such as alterations in the mitochondrial transmembrane potential, generate oxidative stress [76,77]. In addition, in vitro study in SH-SY5Y cells revealed that PKCδ might be responsible for the increased mitochondrial oxidative stress [78]. Moreover, our previous studies suggested that GRe exhibited protective effects against dopaminergic toxicity caused by methamphetamine, an amphetamine analog, via PKCδ-dependent mitochondrial oxidative stress and mitochondrial GPx activity in vitro/in vivo [12,13], indicating that mitochondria might be an intracellular target of GRe.

In this study, DOI treatment caused a constant and significant increase in SOD activity (mitochondria > cytosol) in the hypothalamus of wild-type mice, whereas it did not implicate a simultaneous increase in GPx activity, particularly in the mitochondrial fraction. Increased SOD activity may increase H_2_O_2_ accumulation, facilitate Fenton reaction, and result in irreversible cellular oxidative damage caused by lipid peroxidation (HNE)/protein oxidation/ROS [66]. Our observation of increased oxidative parameters indicates that GPx activity mainly modifies these endpoints rather than SOD. Furthermore, significant elevation of SOD-1 and SOD-2 activity in wild-type mice could be considered by the increased superoxide anion production during the oxidative insult caused by DOI. Thus, it is speculated that PKCδ inhibition or GRe-mediated GPx induction may be accountable for dropping H_2_O_2_ levels. Interestingly, as PKCδ is a well-known redox-sensitive kinase [79], oxidative stress has been shown to upregulate PKCδ activity and facilitate its mitochondrial translocation [43]. Upon mitochondrial translocation, PKCδ induces mitochondrial dysfunction and concurrent oxidative stress, as mentioned above. Thus, PKCδ can be an important mediator of positive feedback amplifiers between oxidative stress and mitochondrial dysfunction [43]. Considering our previous reports [35,54,55] and the present results showing that PKCδ plays a critical role in inducing serotonergic behaviors mediated by 5-HT_1A_ or 5-HT_2A_ receptors, it is suggested that DOI-induced oxidative stress contributes to the induction of serotonergic behaviors, and that GRe-mediated antioxidant potential is important for preventing DOI-induced serotonergic behaviors.

Handy et al. (2009) proposed that mitochondrial functions were regulated by GPx-1 to modulate redox-dependent cellular responses [80]. Moreover, GPx-1 KO increased mitochondrial oxidative stress in association with loss of mitochondrial energy production [81]. It is important to note that the GRe-mediated protective potential with the recovery of mitochondrial function requires inhibition of PKCδ. It is also plausible that GRe protects against DOI-induced mitochondrial impairment through the induction of GPx activity by inhibiting PKCδ. Hence, preservation of mitochondrial transmembrane potential by GRe or inhibition of PKCδ may be critical for the restoration of mitochondrial function.

We observed here that DOI, as a 5-HT_2A_ agonist and an amphetamine analog, facilitated intramitochodrial Ca^2+^ accumulation, which further potentiated mitochondrial oxidative stress and also impaired mitochondrial transmembrane potential. Increased intracellular Ca^2+^ promoted the intramitochondrial Ca^2+^ accumulation when Ca^2+^ influx was more than total Ca^2+^ efflux [82]. This excessive mitochondrial Ca^2+^ may cause uncoupling of mitochondrial electron transport and eventually lead to oxidative damage. We speculate that GRe might attenuate Ca^2+^ influx via inhibition of the mitochondrial translocation of PKCδ, reflecting that GRe primarily restores mitochondrial function.

The effect of DOI on hypothalamic serotonin release has not been well understood. However, previous studies have shown that systemic administration of DOI decreases the extracellular serotonin release [83,84,85], while intra-prefrontal cortical infusion of DOI increases the extracellular serotonin release [86,87] in the prefrontal cortex. Therefore, it remains to be elucidated whether systemic or intrahypothalamic DOI administration significantly affects the serotonin release in the hypothalamus.

5-HT_2A_ receptor-induced serotonergic burden was particularly specific in the hypothalamus [55,88,89]. Consistently, DOI-induced increased expression of c-Fos, a redox-sensitive factor, in the hypothalamus [90], was attenuated by 5-HT_2A_ antagonist (i.e., MDL100907) [90]. Moreover, immunocytochemical and pharmacological data indicates that DOI principally activates 5-HT_2A_ receptors [91], reflecting the importance of modulation of hypothalamic 5-HT_2A_ receptor. We found that serotonergic behaviors triggered by 5-HT_1A_ receptors paralleled those by 5-HT_2A_ receptors [35,54]. However, either one played an opposite role in thermoregulation [92]; for example, activation of 5-HT_1A_ causes hypothermia [35], whereas the stimulation of 5-HT_2A_ receptors activates a hyperthermic response [29,93]. This phenomenon remains to be explored further.

Importantly, serotonin syndrome and hyperthermia can be modulated by therapeutic intervention of the 5-HT_2A_ receptor antagonist, cyproheptadine, and ketanserin [14,29]. In addition, it has been reported that head twitch response is specifically mediated by the 5-HT_2A_ receptor [94,95], and this effect is alleviated by the antagonism of 5-HT_2A_ receptor [36]. Furthermore, in addition to traditional serotonergic behaviors, DOI also induced head twitch response in wild-type mice. Consistently, antagonism of 5-HT_2A_ receptor (i.e., MDL) ameliorated serotonergic impairments, suggesting that 5-HT_2A_ receptors specifically mediate DOI-induced serotonergic impairments. Among different serotonin receptors, 5-HT_2A_ may be associated with complex behaviors [82]. Earlier studies suggested that head twitch response in mice represented a sort of hallucinogenic behavior [95,96], since hallucinogenic agents induce head twitch response in rodents. Thus, DOI is a frequently used pharmacological tool in the head twitch response studies of hallucinogens. Interestingly, mice lacking 5-HT_2A_ receptors do not show a head twitch response to DOI. In contrast, restoration of cortical neuronal 5-HT_2A_ receptors reinstates the DOI potential for inducing head twitch response in the 5-HT_2A_ receptor KO mice [97], reflecting the prerequisite role of 5-HT_2A_ receptors in inducing head twitch response.

In the current study, GRe or rottlerin, or MDL itself without DOI, did not induce significant behavioral or thermal changes (Supplementary Figure S3). Although we did not further examine their effect on the mitochondrial and antioxidant/prooxidant parameters in the absence of DOI in the present study, we have previously reported that GRe or rottlerin alone did not significantly affect the mitochondrial function, mitochondrial/cytosolic antioxidant defense system, or oxidative burden in various brain regions, including the striatum [13,41,46], prefrontal cortex [10], and hippocampus [42]. Nevertheless, the possibility that GRe, rottlerin, or MDL per se exerts its own effect on mitochondrial/cytosolic regulation cannot be ruled out, and it remains to be determined. 

Our findings indicated that GRe attenuated the DOI-induced serotonergic impairments via the recovery from mitochondrial stress by PKCδ inhibition (genetic or pharmacological) in mice. Because GRe or MDL did alter PKCδ KO-mediated protective potentials in mice, it is plausible that PKCδ may be a mechanistic target for DOI-elicited 5-HT_2A_ receptor activation.

In conclusion, our results suggest that PKCδ activation followed by mitochondrial burden might contribute to the serotonergic behaviors induced by DOI, and that the GRe-mediated protective potential regulated by PKCδ inhibition may lead to a novel therapeutic intervention against serotonergic behaviors.

## 4. Materials and Methods 

### 4.1. Preparation of GRe

Mountain-cultivated ginseng (MCG; *Panax ginseng*) was purchased from Pyungchang, Kangwon Province, Republic of Korea, in August 2014. Dried MCG (5 kg) was extracted with 95% ethyl alcohol for 4 h at 78 °C, followed by concentration in vacuum. Ethyl alcohol extract 1320 g was dissolved in 1500 mL of water and extracted with diethyl ether (1500 mL). The water fraction was then evaporated. The water fraction (1214 g) was subjected to column chromatography over Diaion (5 kg) and eluted sequentially with H2O, 30% MeOH, 50% MeOH, 70% MeOH, and 100% MeOH. The 30% MeOH fraction (12.5 g) was chromatographed on ODS (C-18) gel (1 kg) with eluting solvents of 50% MeOH to give four subfractions (F1–F4). The F3 fraction (3.7 g) was further subjected to silica gel column chromatography (500 g, CHCl3: MeOH: H_2_O = 70: 30: 4 v/v) to produce GRe (650 mg) [98].

### 4.2. Animals

All experiments in this study, including the treatment of animals, were performed according to the National Institutes of Health (NIH) Guide for the Humane Care and Use of Laboratory Animals (NIH Publication No. 85–23, 1985; grants.nih.gov/grants/olaw/references/PHSPolicyLabAnimals.pdf; August 2019). All experiments were performed under the Institute for Laboratory Research (ILAR) Guidelines for the Care and Use of Laboratory Animals. Mice were caged in a room maintained at 22 ± 0.5 °C with 12:12 h light/dark cycle and fed ad libitum. All mice were allowed to adapt to the laboratory conditions for at least 2 weeks before the experiments. A breeding pair of PKCδ (±) mice (C57BL/6J background) was gifted by Dr. K. I. Nakayama (Dept. of Molecular Genetics, Medical Institute of Bioregulation, Kyushu University, Fukuoka, Japan) [99]. These mice were further bred into the C57BL/6J background for six generations. Male C57BL/6J background mice were used as wild-type mice in our experiments. DNA was obtained from the tails of mice for the genotyping of wild-type and PKCδ KO mice. Genotyping primers for polymerase chain reaction (PCR) were as follows: 5′-GGAAGAATAAGAAACTGCATCACC-3′ and 5′-GAAGGAGCCAGAACCGAAAG-3′ for endogenous detection, and 5′-GGAAGAATAAGAAA CTGCATCACC-3′ and 5′-TGGGGTGGGATTAG ATAAATG-3′ for mutant detection (Bioneer Corporation, Daejeon, Korea). 

### 4.3. Drug Treatment 

GRe, DOI (Sigma-Aldrich, St. Louis, MO, USA), and MDL11,939 (MDL; Sigma-Aldrich), a 5-HT_2A_ receptor antagonist, were dissolved in sterile saline immediately before use. The PKCδ inhibitor, rottlerin (Biomol Research Laboratories Inc., Plymouth, PA, USA), was dissolved in dimethyl sulfoxide as a stock solution and stored at −20 °C. Rottlerin was further diluted in sterile saline at a concentration of 1 µg/μL immediately before use. 

In the first experiment, male PKCδ KO and wild-type mice, weighing approximately 23 ± 2 g, received a single dose of DOI (2.5 mg/kg, i.p.) or saline. Temporal behavioral patterns and changes in rectal temperature were evaluated for 1 and 2 h, respectively. The application of DOI was based on previous studies [100,101]. In the second experiment, male PKCδ KO and wild-type mice were injected with GRe (10 mg/kg, i.p.) twice a day for 5 d. A single dose of DOI (2.5 mg/kg, i.p.) or saline was administered 2 h after the final treatment with GRe. Additional mice received MDL (3 mg/kg, i.p., 30 min prior to DOI treatment) or rottlerin (3 μg, i.c.v./brain, 6 and 2 h before DOI treatment). The application of GRe, rottlerin, and MDL were based on our previous study [13,35,54]. Serotonergic behaviors and head twitch response were assessed for 30 min post-DOI-treatment. Rectal temperature was measured at 60 min post DOI treatment, and the mice were sacrificed immediately. The brains were dissected, and the hypothalamus was collected instantly, frozen using liquid nitrogen, and stored at −70 °C until analysis. The experimental design is illustrated in Figure 1.

### 4.4. Serotonergic Behaviors 

DOI (2.5 mg/kg, i.p.) was injected after 15 min of acclimatization in a black-painted cage (260 mm × 200 mm × 140 mm). Behaviors linked with rodent serotonin syndrome were recorded in ten (the first experiment) or five (the second experiment) different 1 min time-periods separated by 6-min intervals, starting 5 min post-DOI administration. In each assessment period, the following behaviors were recorded: intermittent behaviors, including forepaw treading, head weaving, backward movement, and forepaw treading (scored on a scale of 0–4; 0 = absent, 1 = present once, 2 = present several times, 3 = present frequently, 4 = present continuously); continuous behaviors, including straub tail, hind limb abduction, low body posture, and tremor (scored on a scale of 0–4; 0 = absent, 1 = perceptible, 2 = weak, 3 = medium, 4 = maximal). Overall serotonergic behavior scores were calculated for each 1 min time-period, and then summed together [35,54,102]. 

### 4.5. Head Twitch Response 

The number of head twitch responses was measured in ten (the first experiment) or five (the second experiment) different 1 min time-periods separated by 6 min intervals, starting 5 min post-DOI-administration. The number of head twitch responses in each 1 min period is displayed, or the total number of head twitch response summed from all the periods is shown [36].

### 4.6. Rectal Temperature 

Rectal temperature was measured by inserting an oil-lubricated thermometer at least 3 cm into the mouse rectum under ambient temperature (21 ± 1 °C). Mice were gently handled to avoid sudden movements. Animals with an unsuccessful attempt of probe insertion were excluded from the group [35,54]. 

### 4.7. Preparation of Cytosolic and Mitochondrial Fraction for Neurochemical and Western Blot Analyses

Hypothalamic tissues were homogenized using ice-cold homogenization buffer comprising 0.5 mM potassium EGTA, 0.25 M sucrose, 10 mM Tris-HCl (pH 7.4), and protease inhibitor cocktail (Sigma-Aldrich) by using a Dounce homogenizer. Homogenates were centrifuged at 2000× *g* for 10 min, and nuclei and unbroken cells were removed. Further, to obtain crude mitochondrial pellets and cytosolic supernatants, the suspension was centrifuged at 12,000× *g* for 15 min. Crude mitochondrial pellets were suspended in 3% Ficoll 400 (Sigma-Aldrich) in Ficoll dilution buffer containing 0.1 mM potassium EGTA, 60 mM sucrose, 10 mM Tris-HCl (pH 7.4), and 0.25 M mannitol. A Ficoll density gradient was formed by pouring crude mitochondrial suspension in 3% Ficoll over 6% Ficoll 400 solution. The suspension was centrifuged at 11,500× *g* for 10 min to obtain purified mitochondrial pellets, further resuspended in a buffer containing protease cocktail (pH 7.4), 210 mM mannitol, 5 mM HEPES, and 70 mM sucrose. For Western blot analysis, 100 μL lysis buffer was added to mitochondrial pellets [103,104]. 

### 4.8. Mitochondrial Preparation for the Measurement of Mitochondrial Membrane Potential and Intramitochondrial Ca^2+^ Level 

Sodium pentobarbital (60 mg/kg) was used to anesthetize the animals and then perfused transcardially with ice-cold homogenization buffer (30 mL) comprising 20 mM HEPES, 250 mM sucrose, and 1 mM EDTA, pH 7.2.

The animals were then decapitated, following which the hypothalami (~1 g) were dissected out, rinsed in homogenization buffer (9 mL), and processed in a tissue homogenizer. The homogenate was centrifuged at 1300× *g* for 10 min, supernatant was removed, and it was centrifuged again at 10,000× *g* for 10 min. Using a hand-held homogenizer, the pellet was then gently resuspended (four strokes) in 30 mL homogenization buffer and centrifuged at 10,000× *g* for 10 min, and the resulting pellet was resuspended and rinsed in an homogenization buffer (EDTA-free). All the centrifugation steps were carried out at 4 °C. The mitochondrial pellet was then resuspended at a final concentration of ~20 mg/mL in 250 mM sucrose, and placed on ice. This process was completed within an hour [13,104,105]. 

### 4.9. Western Blot Analysis 

Sodium dodecyl sulfate-polyacrylamide gel electrophoresis (SDS-PAGE) 8% or 10% was used to separate proteins (20 μg/lane) and followed by transfer onto polyvinylidene difluoride membranes. Then, blocking of membranes was commenced using 5% non-fat milk for 30 min, followed by overnight incubation with primary antibodies against PKCδ (1:10,000; Santa Cruz Biotechnology, Santa Cruz, CA, USA), p-PKCδ at Tyr 311 (1:500; Santa Cruz Biotechnology), COX IV (1:500, Cell Signaling, Danvers, MA, USA), or β-tubulin (1:50,000, Sigma-Aldrich) at 4 °C. They were then incubated with HRP-conjugated secondary anti-rabbit IgG (1:1000, GE Healthcare, Piscataway, NJ, USA) or anti-mouse IgG (1:1000, Sigma-Aldrich) for 2 h. Enhanced chemiluminescence system (ECL Plus^®^, GE Healthcare, Arlington Heights, IL, USA.) was used to visualize membrane, and relative intensities of the bands were measured using PhotoCapt MW (version 10.01 for Windows; Vilber Lourmat, Marne la Vallée, France). Then, all the Western blots were normalized to the intensity of COX IV (mitochondrial fraction) or β-tubulin (cytosolic fraction) [13,104].

### 4.10. Measurement of Mitochondrial Transmembrane Potential

5,5′,6,6′-tetrachloro-1,1′,3,3′-tetraethylbenzimidazolycarbocyanine iodide dye (JC-1; Molecular Probes, Eugene, OR, USA) was used to assess mitochondrial transmembrane potential. This dye exists as a green-fluorescent monomer at low membrane potential but reversibly forms red-fluorescent “J-aggregates” at polarized mitochondrial potentials. Briefly, 250 μg aliquots of isolated mitochondrial protein from hypothalamic tissues were suspended in respiration buffer comprising 20 mM HEPES, 2.5 mM inorganic phosphates (pH 7.2), 250 mM sucrose, 2 mM MgCl_2_, and 10 mM succinate (5 mM glutamate and 2.5 mM maleate gave similar results in all paradigms) in a final volume of 200 μL. The energized mitochondria were then incubated with 10 μM JC-1 for 30 min at 37 °C, and fluorescence was measured using a fluorescence plate reader (Molecular Devices Inc., Sunnyvale, CA, USA). Mitochondrial polarization was measured by taking the emission ratio from 590 nm to 535 nm with excitation at 490 nm [13,105,106,107,108]. 

### 4.11. Measurement of Intramitochondrial Ca^2+^ Levels

Mitochondrial fractions (250 μg) were incubated at 37 °C with Rhod-2-AM (5 μM; Molecular Probes) for 1 h, followed by washing with Ca^2+^-free Locke’s solution (3–4 times). This reduced form of Rhod-2-AM is a colorless, non-fluorescent dye with a net positive charge, which promotes sequestration into the mitochondria, whereas dye oxidized in the mitochondria and cleaved AM ester are trapped inside the mitochondria. Fluorescence plate reader (Molecular Devices Inc.) was used to measure fluorescence with an excitation wavelength of 549 nm and emission wavelengths of 581 nm [13,108,109,110].

### 4.12. Measurement of Complex I Activity 

Isolated mitochondrial samples were added to a reaction mixture comprising 3.5 mg/mL bovine serum albumin, 25 mM potassium phosphate buffer (pH 7.8), 1 μM antimycin A, 70 μM decylubiquinone, and 60 μM 2,6-dichloroindophenol, and the reaction mixture was incubated at 37 °C for 3 min. NADH (0.2 mM) was added, and absorbance was measured at 600 nm for 4 min at intervals of 60 s using a microplate reader (Spectra Max Plus 384, Molecular Devices Inc., Sunnyvale, CA, USA). Rotenone (1 μM) was then added, and the absorbance was measured again at 600 nm for 4 min at intervals of 60 s. One unit of complex I activity is defined as 1 μmoL 2,6-dichloroindophenol reduced per minute, and it was calculated based on the extinction coefficient for 2,6-dichloroindophenol (19.1 mM^−1^ cm^−1^). The results have been expressed as a percentage of the control group [10,108,111]. 

### 4.13. Measurement of Mitochondrial Complex II Activity

Reaction mixtures contained 2 mM EDTA, 80 mM potassium phosphate buffer (pH 7.8), 80 μM 2,6-dichloroindophenol, 1 mg/mL bovine serum albumin, 50 μM decylubiquinone, 3 μM rotenone, and 1 μM antimycin A. These were incubated for 10 min at 37 °C. Potassium cyanide (KCN) (0.3 mM) and succinate (10 mM) were added to start the reaction. Absorbance at 600 nm was recorded at 37 °C for 5 min at intervals of 1 min, using a microplate reader (Spectra Max Plus 384, Molecular Devices Inc., Sunnyvale, CA, USA). One unit of complex II activity is defined as the reduction of 1 μmol 2,6-dichloroindophenol per minute. Calculation of activity was based on the extinction coefficient of 2,6-dichloroindophenol (19.1 mM^−1^ cm^−1^) [10,108,111].

### 4.14. Determination of ROS 

Cytosolic and mitochondrial fractions were incubated with 5 μM 2′,7′-dichlorofluorescein diacetate (DCFH-DA, Molecular Probes) for 15 min at 37 °C. The fluorescence intensity was measured at an excitation and emission wavelength of 488 nm and 528 nm, respectively, using a fluorescence microplate reader (Molecular Devices Inc.) [104,112].

### 4.15. Determination of HNE

OxiSelectTM HNE adduct ELISA kit (Cell Biolabs, Inc., San Diego, CA, USA) was used to determine the quantity of lipid peroxidation by assessing the level of 4-hydroxynonenal (HNE), according to the manufacturer’s instruction. Cytosolic and mitochondrial fractions (100 μL) at a protein concentration of 10 μg/mL were incubated in a 96-well protein binding plate at 4 °C overnight. HNE adducts in each well were labeled with HNE antibody after the protein adsorption, followed by HRP-conjugated secondary antibody. Substrate solution was then added to perform colorimetric development. Absorbance was recorded at 450 nm using a microplate reader (Molecular Devices Inc.), and the standard curve of HNE-BSA was used to calculate the amount of HNE adduct in each sample.

### 4.16. Determination of Protein Carbonyl

Protein oxidation was determined by analyzing the content of protein carbonyl groups using a 2,4-dinitrophenylhydrazine (DNPH)-labeling procedure [113]. DNPH-labeled protein was detected using a microplate reader [10,13,104,108], and the results were represented as nmol of DNPH incorporated/mg protein based on the extinction coefficient for aliphatic hydrazones (21 mM^−1^ cm^−1^). BCA Protein Assay kit (Thermo Scientific) was used to measure protein concentration.

### 4.17. Determination of SOD

The reaction mixture containing 30 μM cytochrome c, 70 mM potassium phosphate buffer (pH 7.8), 150 μM xanthine, and cytosolic or mitochondrial preparations in phosphate buffer was diluted 10-fold with PBS to a final volume of 3 mL. The reaction was initiated by adding 50 units xanthine oxidase (10 μL), and absorbance was measured at 550 nm by microplate reader (Molecular Devices Inc.). One unit of SOD is defined as the quantity required to inhibit the rate of cytochrome c reduction by 50%. Total SOD was measured by adding KCN (10 μM) to the medium to inhibit cytochrome oxidase activity. To estimate mitochondrial Mn-SOD activity, Cu, Zn-SOD activity was abolished by adding KCN (1 mM) to the mixture. Cu, Zn-SOD activity was calculated by deducting the Mn-SOD activity from the total SOD activity [10,13].

### 4.18. Determination of GPx

The incubation mixtures contained 0.2 mM NADPH, 2 mM reduced glutathione, and 1.4 IU glutathione reductase in 0.05 M potassium phosphate buffer, pH 7.0. Reactions were initiated by simultaneous addition of cytosolic or mitochondrial fractions (0.3–0.8 mg protein) and 0.25 mM cumene hydroperoxide. Microplate reader (Molecular Devices Inc.) was used to measure absorbance at 340 nm at room temperature. The reaction rate at 340 nm was determined using the NADPH extinction coefficient (6.22 mM^−1^ cm^−1^), and GPx activity was expressed as nmol NADPH oxidized per minute per milligram protein at 25 °C [10,13,114].

### 4.19. Statistical Analyses

In the present study, data analysis was accomplished by one-way analysis of variance (ANOVA) with post-hoc Fisher’s least significant difference (LSD) pairwise comparison tests using IBM SPSS ver. 24.0 (IBM Corp., Armonk, NY, USA). Temporal changes in serotonergic behaviors, head twitch response, and rectal temperature were analyzed using two-way ANOVA with post-hoc Fisher’s LSD pairwise comparison tests, and *p*-values < 0.05 reflected statistical significance.

## Data Availability

The data used and/or analyzed during the current study are available from the corresponding author on reasonable request.

## Supplementary Materials

The following are available online at https://www.mdpi.com/article/10.3390/ijms22137219/s1, Figure S1. Western blot data of Figure 2 in main text. Figure S2. Effect of GRe or 5-HT_2A_ receptor antagonist MDL on the DOI-induced serotonergic behaviors: head weaving (A), forepaw treading (B), hind-limb abduction (C), straub tail (D), tremor (E), and low body posture (F) in wild-type and PKCδ KO mice. Supplementary Figure S3. Effect of GRe, rottlerin, or MDL alone on overall serotonergic behavioral score (A), head twitch response (B), and rectal temperature (C) in wild-type and PKCδ KO mice.
