# Supplementary material for: Ginsenoside Re Protects against Serotonergic Behaviors Evoked by 2,5-Dimethoxy-4-iodo-amphetamine in Mice via Inhibition of PKCδ-Mediated Mitochondrial Dysfunction
Source: Int J Mol Sci. 2021 Jul 5;22(13):7219. doi: 10.3390/ijms22137219 (PMC8268959; doi:10.3390/ijms22137219)
Supplement: Supplementary file 1 [file ijms-22-07219-s001.zip › ijms-1227411-supplementary.pdf]

Supplementary Information

Article

Ginsenoside Re protects against serotonergic behaviors evoked by 2,5-dimethoxy-4-iodoamphetamine in mice via inhibition of PKCδ-mediated mitochondrial dysfunction

Eun-Joo Shin[1,#], Ji Hoon Jeong[2,#], Bao-Trong Nguyen[1], Naveen Sharma[1,2], Seung-Yeol Nah[3], Yoon Hee Chung[4], Yi Lee[5], Jae Kyung Byun[6], Toshitaka Nabeshima[7], Sung Kwon Ko[8,*], Hyoung-Chun Kim[1,*]

[1]Neuropsychopharmacology and Toxicology Program, College of Pharmacy, Kangwon National University, Chunchon 24341, South Korea

[2]Department of Global Innovative Drugs, Graduate School of Chung-Ang University, College of Medicine, Chung-Ang University, Seoul 06974, Republic of Korea

[3]Ginsentology Research Laboratory and Department of Physiology, College of Veterinary Medicine and Bio/Molecular Informatics Center, Konkuk University, Seoul 05029, Republic of Korea

[4]Department of Anatomy, College of Medicine, Chung-Ang University, Seoul 06974, Republic of Korea

[5]Department of Industrial Plant Science & Technology, Chungbuk National University, Chungju 28644, Republic of Korea

[6]Korea Society of Forest Environmental Research, Namyanju 12106, Republic of Korea

[7]Advanced Diagnostic System Research Laboratory, Fujita Health University Graduate School of Health Science, Toyoake 470-1192, Japan

[8]Department of Oriental Medical Food and Nutrition, Semyung University, Jecheon, 27136, Republic of Korea.

#First two authors equally contributed to this work.

*Corresponding author: Hyoung-Chun Kim, Neuropsychopharmacology & Toxicology Program, College of Pharmacy, Kangwon National University, Chunchon 24341, Republic of Korea. Tel: +82-33-250-6917; Fax: +82-33-259-5631; E-mail: kimhc@kangwon.ac.kr

or

Sung Kwon Ko, Department of Oriental Medical Food and Nutrition, Semyung University, Jecheon, 27136, Republic of Korea. E-mail: skko@semyung.ac.kr

1. Supplementary Results

1.1. Western blot data of Figure 2 main text.

Data of original western blot membrane (Supplementary Fig. S1A and S1B) support Fig. 2 and 2.1 of main text.

1.2. Effect of GRe or MDL11939 (MDL) on 5-HT$_{2A}$ agonist DOI-induced serotonergic behaviors in wild-type and PKCδ KO mice

As shown in Supplementary Fig. S2, DOI treatment consistently and significantly induced ($P < 0.01$ vs. Saline/Saline) a respective serotonergic behavior (i.e., head weaving, forepaw treading, hind limb abduction, straub tail, tremor, low body posture, and head-twitch response in *wild-type* mice, and DOI-induced serotonergic behaviors were consistently and much less pronounced ($P < 0.05$ vs. Saline/DOI/*wild-type*) in *PKCδ KO* mice. GRe, rottlerin, or MDL consistently and significantly attenuated ($P < 0.05$ vs. Saline/DOI) the respective serotonergic behavior induced by DOI in *wild-type* mice. GRe or MDL did not show any additional effects on *PKCδ KO*-mediated protective potential against DOI-induced serotonergic behaviors in mice (Supplementary Fig. S2).

1.3. *Effect of GRe, Rottlerin, or MDL alone on overall serotonergic behavioral score, head twitch response, and rectal temperature in wild-type and PKCδ KO mice.*

As shown in Supplementary Fig. S3, we found that GRe, Rottlerin, or MDL alone did not significantly alter overall serotonergic behavior (A), head twitch response (B), and rectal temperature (C).

A. Cytosolic Fraction

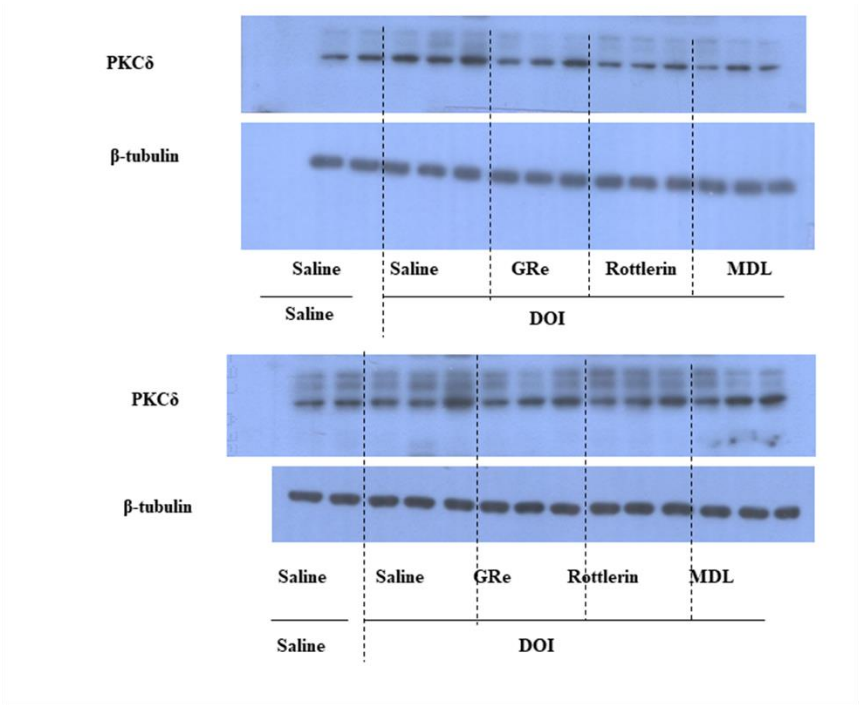

B. Mitochondrial Fraction

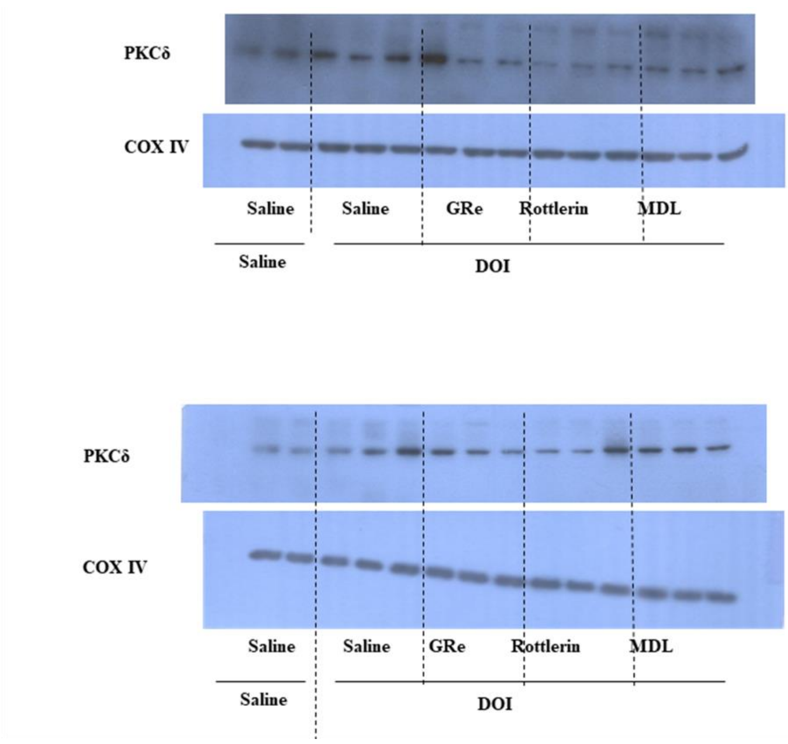

Supplementary Figure S1. Western blot data of Figure 2 main text.

A.

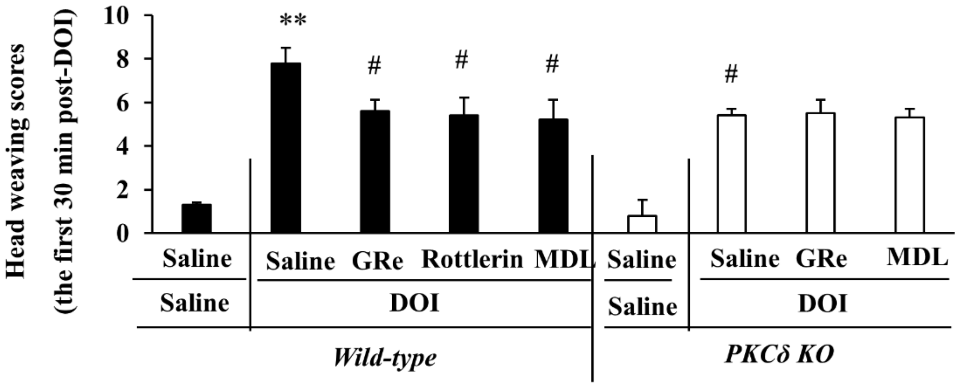
Head weaving scores
(the first 30 min post-DOI)
10
8
6
4
2
0
**
#
#
#
#
Saline
Saline
GRe
Rottlerin
MDL
Saline
Saline
GRe
MDL
Saline
DOI
Saline
DOI
Wild-type
PKCδ KO

B.

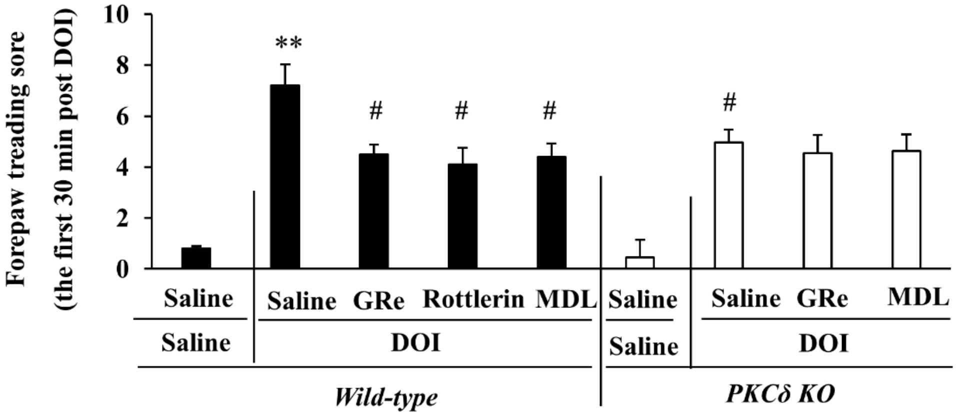
Forepaw treading sore
(the first 30 min post DOI)
10
8
6
4
2
0
**
#
#
#
#
Saline
Saline
GRe
Rottlerin
MDL
Saline
Saline
GRe
MDL
Saline
DOI
Saline
DOI
Wild-type
PKCδ KO

C.

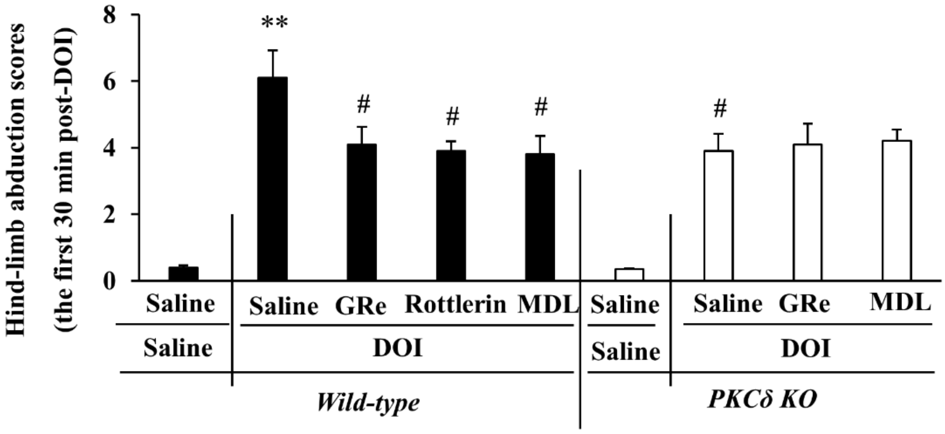
Hind-limb abduction scores
(the first 30 min post-DOI)
8
6
4
2
0
**
#
#
#
#
Saline
Saline
Saline
GRe
Rottlerin
MDL
DOI
Wild-type
Saline
Saline
Saline
GRe
MDL
DOI
PKCδ KO

D.

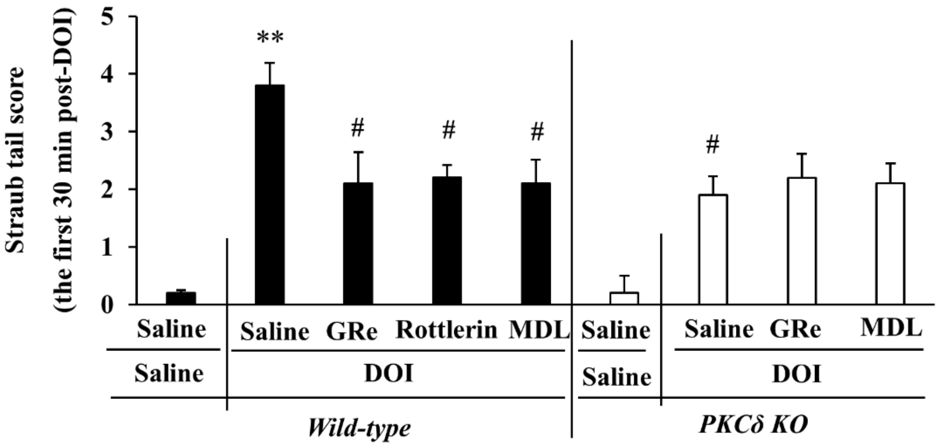
Straub tail score
(the first 30 min post-DOI)
5
4
3
2
1
0
**
#
#
#
#
Saline
Saline
Saline
GRe
Rottlerin
MDL
DOI
Wild-type
Saline
Saline
Saline
GRe
MDL
DOI
PKCδ KO

E.

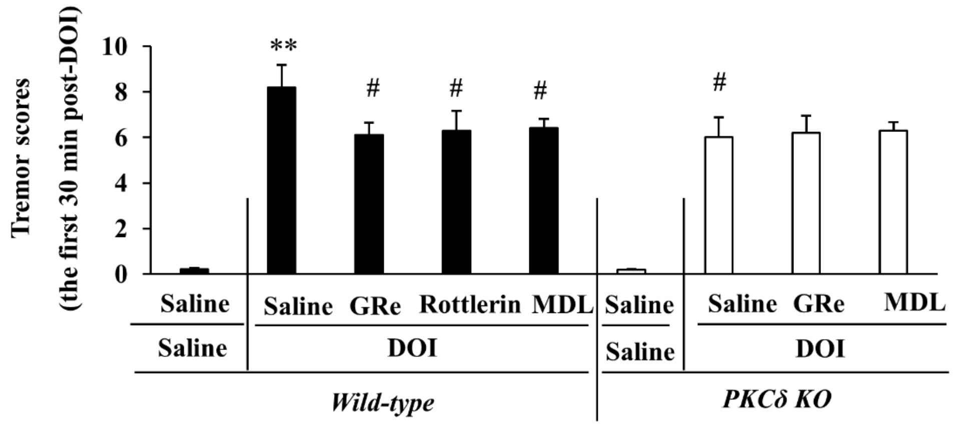

F.

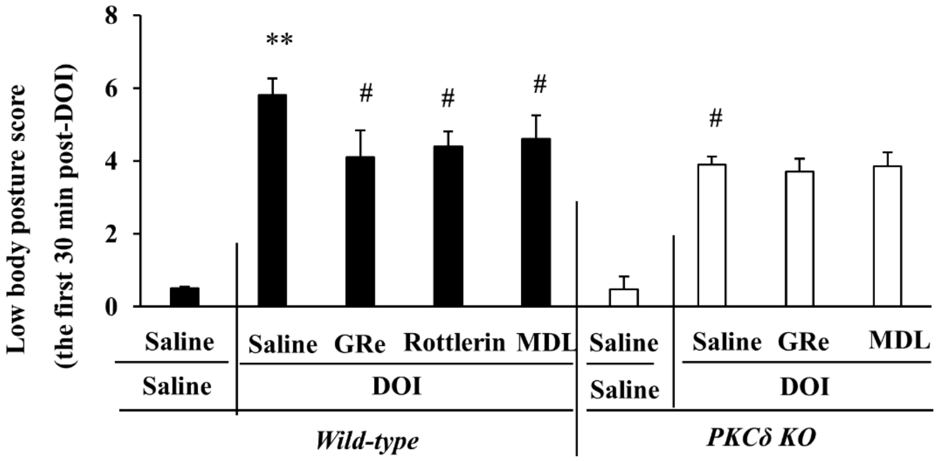

Supplementary Figure S2. Effect of GRe or 5-HT_{2A} receptor antagonist MDL on the DOI-induced serotonergic behaviors; head weaving (A), forepaw treading (B), hind-limb abduction (C), straub tail (D), tremor (E), and low body posture (F) in *wild-type* and *PKCδ KO* mice. GRe, ginsenoside Re; MDL, MDL11939; DOI, 2,5-dimethoxy-4-iodoamphetamine; *PKCδ KO*, *PKCδ knockout* mice. Each value is the mean ± SEM of six animals. **P < 0.01 vs. Saline/Saline/*Wild-type*. $^{\#}$P < 0.05 vs. Saline/DOI/*Wild-type* (one-way ANOVA followed by Fisher's LSD pairwise comparisons).

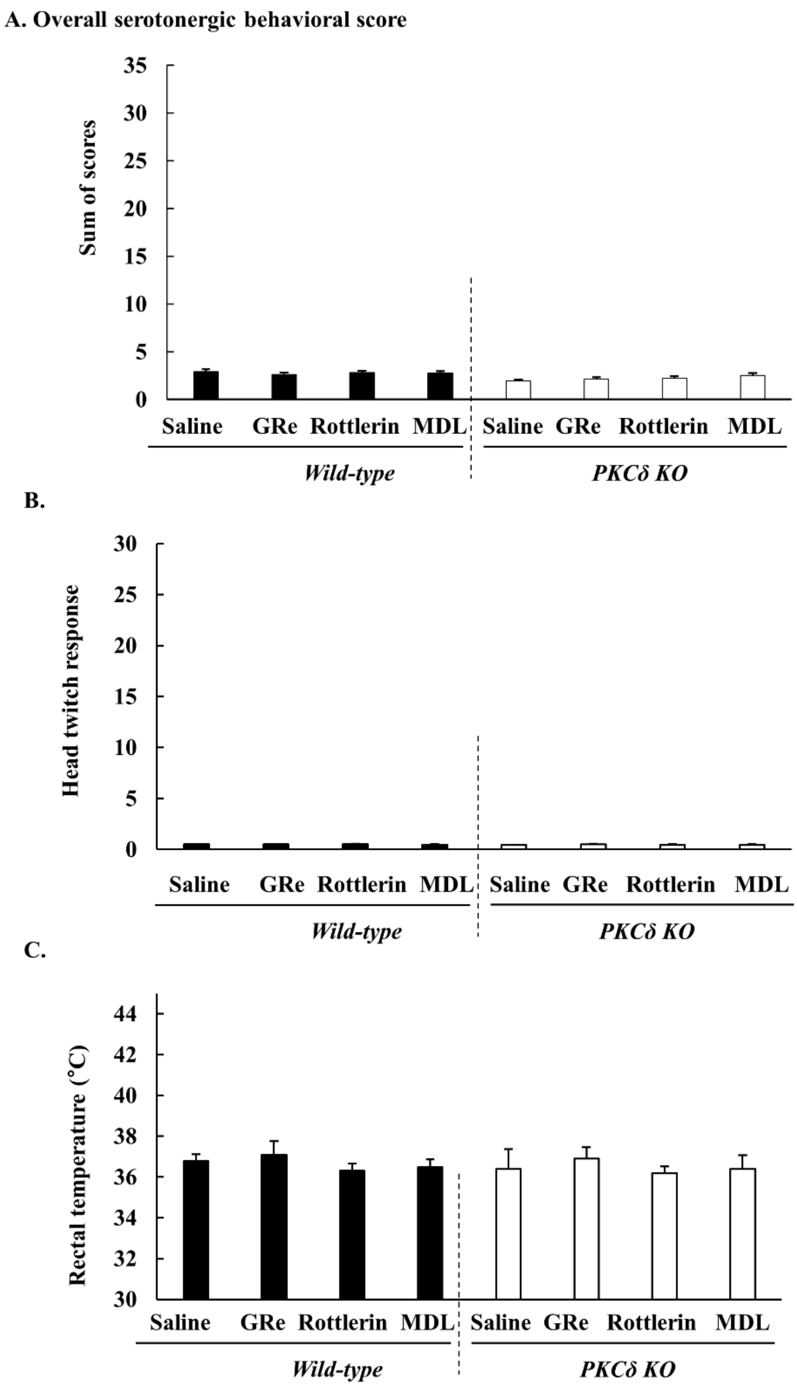

Supplementary Figure S3. Effect of GRe, Rottlerin, or MDL alone on overall serotonergic behavioral score, head twitch response, and rectal temperature in *wild-type* and *PKCδ* KO mice. Effects on overall serotonergic behavior score within the first 30 min (A). Effects on head twitch response within the first 30 min (B). Effects on the rectal temperature after 60 min of

administration (C). Data are expressed as the mean ± SEM (6 animals / group). GRe, ginsenoside Re; MDL, MDL11939; DOI, 2,5-dimethoxy-4-iodo-amphetamine; *PKCδ* KO, *PKCδ* knockout mice.
